# Neural patterns elicited by sentence processing uniquely characterize typical development, SLI recovery, and SLI persistence

**DOI:** 10.1186/s11689-017-9201-1

**Published:** 2017-06-14

**Authors:** Eileen Haebig, Christine Weber, Laurence B. Leonard, Patricia Deevy, J. Bruce Tomblin

**Affiliations:** 1 Purdue University, West Lafayette, IN USA; 2 University of Iowa, Iowa City, IA USA

**Keywords:** Specific language impairment, Language trajectories, Sentence processing, Event-related brain potentials, N400, P600

## Abstract

**Background:**

A substantial amount of work has examined language abilities in young children with specific language impairment (SLI); however, our understanding of the developmental trajectory of language impairment is limited. Along with studying the behavioral changes that occur across development, it is important to examine the neural indices of language processing for children with different language trajectories. The current study sought to examine behavioral and neural bases of language processing in adolescents showing three different trajectories: those with normal language development (NL), those exhibiting persistent SLI (SLI-Persistent), and those with a history of SLI who appear to have recovered (SLI-Recovered).

**Methods:**

Through a sentence judgment task, we examined semantic and syntactic processing. Adolescents judged whether or not each sentence was semantically and syntactically correct. Stimuli consisted of naturally spoken sentences that were either correct, contained a semantic verb error, or contained a syntactic verb agreement error. Verb agreement errors consisted of omission and commission violations of the third-person singular -*s*. Behavioral button-press responses and electroencephalographic recordings were collected. Behavioral judgments and mean amplitude of the N400 and P600 components were examined.

**Results:**

Adolescents in the SLI-Persistent group had lower sentence judgment accuracy overall, relative to the NL and SLI-Recovered groups. Accuracy in judging omission and commission syntactic errors were marginally different, with marginally lower accuracy for commission errors. All groups demonstrated an N400 component elicited by semantic violations. However, adolescents in the SLI-Persistent group demonstrated a less robust P600 component for syntactic violations. Furthermore, adolescents in the SLI-Recovered group exhibited a similar neural profile to the NL group for the semantic and syntactic omission violations. However, a unique profile with initial negativity was observed in the SLI-Recovered group in the commission violation condition.

**Conclusions:**

Adolescents with persistent language impairment continue to demonstrate delays in language processing at the behavioral and neural levels. Conversely, the adolescents in the SLI-Recovered group appear to have made gains in language processing skills to overcome their initial impairments. However, our findings suggest that the adolescents in the SLI-Recovered group may have compensatory processing strategies for some aspects of language, as evidenced by a unique event-related potential profile.

## Background

With some exceptions, studies of children with specific language impairment (SLI) have focused on earlier stages of development. Consequently, we have limited knowledge of how the language abilities of these children change over time [7, 80]. As such, it is important to examine later developmental periods to enhance our understanding of the trajectory of SLI. This seems especially crucial because some children initially diagnosed with SLI appear to achieve close-to-normal language skills at a later age, whereas others continue to exhibit a significant language deficit.

Beyond behavioral changes, it is important to study neural modifications throughout development. We have limited understanding of the link between the brain and behavior, especially in individuals with atypical development. Karmiloff-Smith [28] suggested that children with disorders may demonstrate behaviors that resemble those of typically developing children; however, despite overlap in behavioral performance, the underlying neural correlates may differ. In addition, it is possible that children with SLI demonstrate delays in early neuromaturation that could lead to delayed language development [39]. Therefore, it is important to examine whether there is neural evidence of language deficits or delays later in development [80]. This study examined behavioral and neural bases of language processing in adolescents with SLI who demonstrate persistent language impairment and those who appear to have recovered.

### SLI across development

Children with SLI have a core deficit in language in the absence of frank neurological or genetic disorders, intellectual disability, or hearing impairment [32, 75]. Both lexical-semantic deficits and grammatical deficits in the expressive and receptive domains have been documented in these children [34, 62, 63, 70]. In addition to linguistic deficits, however, children with SLI have nonlinguistic impairments such as working memory deficits and limitations in processing speed [12, 35, 45, 54].

As children with SLI age, hallmark features of grammatical deficits become less apparent. Some children with SLI no longer meet diagnostic criteria for an SLI classification by late childhood or adolescence; however, language deficits in others can persist [7, 63]. In addition to changes in diagnostic classifications across development, it has been suggested that the pattern of language difficulties may shift with maturation [23]. It is therefore important to explore language profiles in children with persistent language impairment and children with a history of language impairment who appear to have overcome their deficits in language.

#### Children with a history of SLI

Careful examination of abilities in children with a history of SLI is particularly important given that, despite growth in language abilities, many children continue to experience academic struggles including reading and math difficulties [5, 6, 13]. In fact, it has been suggested that children who have a history of SLI but no longer meet diagnostic criteria often fit into an “illusory recovered” group [66]. Thus, additional research is needed to identify areas of processing in which adolescents with a history of SLI demonstrate subclinical residual vulnerability.

Some initial work has been devoted to the study of children with a history of SLI. Hesketh and Conti-Ramsden [23] examined predictive profiles of sentence repetition abilities. Grammatical and phonological working memory abilities were found to predict sentence repetition in children with a history of SLI; however, for typically developing children, only grammatical abilities were predictive. Hesketh and Conti-Ramsden suggested that children with a history of SLI may not have sufficiently secure language knowledge (i.e., word representations, knowledge of predictable sentence structures) to facilitate chunking linguistic information for sentence repetition. As such, these children also must rely on phonological working memory abilities. Borovsky and colleagues [4] also found subtle language weaknesses in adolescents with a history of SLI, relative to typically developing adolescents. Using an eye-gaze study to examine real-time lexical processing at the sentence level, Borovsky and colleagues found that adolescents with a history of SLI were less likely to look at images that could be relevant with an alternative sentence interpretation (e.g., action-related images), suggesting difficulties with lexical integration [4].

Lastly, Purdy and colleagues found that children with a history of SLI were able to identify verb agreement violations in simple, local-dependency sentences [57]. However, when sentences were more taxing, with long-distance finiteness errors, children with a history of SLI had lower grammaticality judgment accuracy and less robust event-related potential (ERP) components indexing grammatical language processing. Therefore, although children with a history of SLI have gained linguistic skills, the current literature suggests that language processing remains divergent from typically developing peers at the behavioral and neural levels. The current study aims to contribute additional information about the neural and behavioral profiles of language processing in children with a history of SLI who appear to recover, and to compare these profiles with children who continue to meet the diagnostic criteria for SLI.

### Lexical processing and the importance of examining verbs

In addition to syntactic deficits, previous work has identified lexical-semantic deficits in children with SLI [64, 70]. These children are believed to have a reduced breadth and depth of word knowledge [59, 70]. Importantly, findings of lexical-semantic weakness align with accounts indicating that children with SLI have difficulties processing verbs [2, 4, 32]. Despite this, there is limited research examining the lexical-semantic processing of verbs by children with SLI; thus, additional work specifically examining lexical-semantic processing of verbs is needed.

Furthermore, relatively little is known about online lexical processing at the sentence level [4]. Although several studies have examined sentence processing in children with SLI, the majority of this work has examined morphosyntactic aspects of sentence processing rather than semantic (e.g., [61, 83]). Additionally, many of these studies have relied on button-press responses that reflect behavioral indices of language processing that typically occur at the end of a sentence, after the majority of the information in the sentence has been presented. Conflicts in semantic processing may resolve quickly and therefore may not be identified on behavioral tasks. A benefit of using electrophysiological data is that it can capture indices of lexical integration at or shortly after lexical-semantic verb violations.

#### ERP components of lexical processing

When examining neural indices of language processing, the N400 component has been widely used as a means of assessing lexical integration [30, 31]. The N400 is associated with an increase in negative polarity that typically peaks around 400 ms after a semantically anomalous word is presented (e.g., “I like my coffee with cream and *turtle*.”). This component is typically observed at electrode sites over centroparietal regions of the brain [42].

In a sentence-level semantic processing task, Neville and colleagues measured the N400 component in 8- to 10-year-old children [46]. Neville and colleagues found that children with SLI displayed larger N400s over the occipital regions when reading open-class words (e.g., nouns, adjectives, verbs). It was suggested that the larger N400 amplitudes indicated greater effort to integrate semantically rich word classes, relative to typically developing peers. Importantly, Neville et al. [46] also measured responses to anomalous nouns at the end of the printed sentences. They found that not only did children with SLI have reduced behavioral accuracy in identifying anomalous words, but they also displayed larger amplitudes for the N400 elicited by anomalous nouns, relative to the typically developing peers. Overall, Neville and colleagues proposed that the relatively larger N400 amplitudes in the children with SLI indexed greater effort for lexical integration and increased reliance on context for word recognition. In contrast, Fonteneau and van der Lely [15] examined the N400 elicited by anomalous nouns in the middle of sentences that were presented auditorily (e.g., “Sally cooks the *car* in the kitchen.”) to children between the ages of 10 and 21 years. Children with SLI, with particularly weak grammatical skills, were found to show similar N400 responses to age-matched controls. The authors concluded that, in this older and more specific sample of children with SLI, ERP findings suggested that semantic processing is relatively intact.

Lastly, two studies have examined semantic violations of verbs using ERPs. Sabisch and colleagues [64] played recorded sentences with sentence final verbs that were anomalous or semantically correct to German-speaking children between 9 and 10 years of age. Typically developing children exhibited larger N400 amplitudes for anomalous verbs relative to control verbs. In contrast, children with SLI did not have differences between the conditions. However, the children with SLI had relatively large N400 amplitudes for both correct and anomalous verbs, which indicated that children with SLI have weak representations of verbs and as a result have difficulties integrating verb meanings within the context of the broader sentence. Weber-Fox and colleagues [80] also examined verb processing in adolescents with typical language abilities or with SLI. Adolescents with SLI had lower accuracy in identifying anomalous verbs, relative to their peers, aligning with previous research that children with SLI may have weak semantic representations of verbs [32, 64]. However, there were no group differences in the mean amplitude of the N400 component; both groups demonstrated larger N400 effects in response to anomalous verbs than to control verbs. Weber-Fox and colleagues suggested that more robust N400 effects and possibly group differences may have been seen had they used more frank semantic violations instead of open-class semantic violations that may be more easily integrated into a sentence. Therefore, given the mixed findings in the current literature, additional work is needed to examine verb processing in children with different language trajectories using both behavioral and neural measures. In addition to providing a careful examination of semantic processing of verbs, the current study provides unique insight into processing in adolescents with a history of SLI who appear to have recovered.

### Verb agreement

As previously noted, it is well documented that children with SLI have hallmark deficits in verb agreement (e.g., [33, 60]). With age, language deficits become more subtle across a range of language areas and deficits in grammatical morphology become less salient. Nevertheless, studies have demonstrated that school-age children and adolescents with SLI continue to have poorer performance relative to their age-matched peers on grammaticality judgment tasks that include verb agreement errors [45, 58, 61]. Two types of verb agreement violations that have been studied are errors of omission and errors of commission. In omission errors, a necessary bound morpheme is omitted (e.g., dog play vs. dog plays). Conversely, a bound morpheme is inappropriately inserted during commission errors (e.g., babies cries vs. babies cry). Although commission errors are rarely produced in spoken language in children with SLI, Redmond and Rice [58] found that school-age children with SLI accepted commission errors within complex sentences in a sentence judgment task more often than typically developing children.

Leonard et al. [36] also explored effects of omission and commission errors on sentence processing in adolescents with SLI. Leonard et al. carefully controlled for potential confounds of high metalinguistic and working memory demands by creating a word monitoring task. In this task, adolescents were asked to identify a target word as soon as they heard it in a sentence. Importantly, some of the sentences were grammatically correct, but other sentences contained an omission or commission error in the word preceding the target. If the omission or commission error was detected, the response to the subsequent target word was expected to be slower. As predicted, the typically developing children had slower reaction times when identifying the target word if a grammatical commission or omission error occurred before the target word. However, children with SLI did not have slower reaction times when an omission error preceded the target; this pattern was more apparent for past tense -*ed* omissions, relative to third-person singular -*s* omissions. Leonard and colleagues suggested that adolescents with language impairment may continue to have subtle difficulties with verb agreement in later points of development.

#### ERP components of syntactic processing

Syntactic violations are associated with a late positivity, the P600 component, which is thought to indicate syntactic reanalysis that occurs after identifying a syntactic violation or ambiguity [18, 50, 51]. Friederici [16] also suggested that an earlier component generally referred to as an anterior negativity (AN; sometimes also referred to as the left anterior negativity (LAN) or the early left anterior negativity (ELAN)) marks the initial detection of a morphosyntactic error between 100 and 500 ms after the violation. However, the anterior negativity has not been consistently elicited [72]. It also has been proposed that earlier negativities observed in stimuli with long-distance dependencies may reflect the increased memory load associated with holding incomplete syntactic dependencies in memory [55].

Studies examining neural components associated with syntactic errors in children with SLI have reported mixed findings. Fonteneau and van der Lely [15] found that violations of syntactic dependencies, for example in wh- questions, elicited a P600 response in children with typical development and SLI. However, children with SLI with particularly weak syntactic skills did not display the AN component. Instead, the children with SLI had a later posterior negativity that was similar to the N400 component. In contrast, Sabisch and colleagues observed the P600 component but also the AN component when children were presented with word category violations and prosodic incongruities [65].

Weber-Fox and colleagues [80] specifically examined neural components following verb agreement violations that included omission and commission errors of the third-person singular -*s*. They found that verb agreement violations elicited the AN component in adolescents with typical development and adolescents with SLI. However, the P600 component was only observed in the typically developing group. In addition, as previously noted, Purdy and colleagues [57] found that local third-person singular -*s* commission violations elicited the AN and P600 components in school-age typically developing children and children with a history of SLI. Long-distance commission violations elicited a robust mean amplitude of the P600 for the typically developing group. The children with a history of SLI, however, displayed a P600 that was delayed, was shortened in duration, and had reduced amplitude. The AN component was not observed for the long-distance commission violations for either group. Additional work is required to form a more complete understanding of ERP components elicited by verb agreement violations in children with varying trajectories of language development. Furthermore, it is important to examine whether the neural profiles differ for omission and commission verb agreement errors, given that only omission errors are commonly seen in the productions of children with SLI.

### The current study

The current study examined the neural indices of lexical-semantic and syntactic processing in adolescents with different language trajectories. This work contributes information about language processing in children with language impairment at later points of development to provide a more complete picture of the developmental course of language impairments. Additionally, this work contributes insights into the ways in which children with atypical language development potentially rely on compensatory processing strategies to overcome early language deficits. In addition to examining semantic processing of verbs, we specifically examined two types of verb agreement errors (i.e., omission and commission violations) during language processing using behavioral and neural methods, which can allow for direct comparisons of linguistic forms that are more apparent in language impairments than others. Furthermore, this study contributes needed information about the neural profiles of adolescents with normal language development, a history of language impairment, and persistent language impairment, providing a more comprehensive picture of language trajectories. As such, the current study has the potential to provide insight into the underlying neural processes that mediate recovery of language impairments. Our specific research questions were:

1. Does behavioral performance on sentence processing differ across adolescents with different language trajectories?
2. Do neural signals differentiate adolescents with different language trajectories during a sentence processing task?
3. Does verb agreement error type influence group differences?

Given previous findings, we predicted that adolescents with persistent SLI (SLI-Persistent) and adolescents with a history of SLI who had normal language abilities later in development (SLI-Recovered) would have lower accuracy on the sentence judgment task than adolescents with no history of language impairment. Additionally, we predicted that some neural profiles would differentiate the groups. First, given the age of our participants, we predicted that all three groups would demonstrate an N400 effect in response to lexical-semantic verb violations. However, if differences were observed, we would predict that the adolescents in the SLI-Persistent group may present with a less mature neural response to the lexical-semantic violations, following Locke’s [39] suggestion that children with SLI experience a persistent lag in neuromaturation. We also predicted that adolescents with SLI would demonstrate a less robust P600 component following syntactic violations (commission and omission violations). Furthermore, if the sentence judgment task was sufficiently difficult, we predicted that adolescents in the SLI-Recovered group would also demonstrate a different neural profile relative to adolescents with typical language development in response to verb agreement violations. Lastly, we expected to see differences in judgment accuracy for errors of omission and errors of commission. Specifically, we expected accuracy to be higher for commission errors than for omission errors. Given the limited previous work examining omission and commission errors separately, our predictions were tentative, but we expected the commission verb agreement violations to elicit a robust P600 component because it is not a common error that individuals produce and therefore may be more salient.

## Methods

### Participants

Participants were 52 adolescents who participated in a larger longitudinal study on the prevalence of SLI [75]. In the Tomblin et al. study, children completed standardized cognitive and language tests in kindergarten, second grade, fourth grade, and eighth grade. Test scores determined group classification for each visit. The group classifications from the parent grant were used to assign participants in the current study to one of three categories: Normal Language (NL), SLI-Recovered (SLI-R), and SLI-Persistent (SLI-P).

Adolescents in the NL group (*n* = 18) had no history of language impairment across the larger study’s four time points. Adolescents in the SLI-Recovered group (*n* = 15) had a history of SLI in kindergarten and/or second grade, but normal language abilities in fourth and eighth grades. Lastly, adolescents in the SLI-Persistent group (*n* = 19) received a classification of SLI in eighth grade and a status of SLI or nonspecific language impairment during at least two of the three previous grades. Across the four visits during the longitudinal study, the children in the NL had significantly higher language composite scores than the SLI-Recovered and SLI-Persistent groups. Additionally, the children in the SLI-Recovered group had significantly higher language composite scores relative to the SLI-Persistent group. However, as would be expected given the group classifications, the difference in language abilities between the SLI-Recovered and SLI-Persistent groups grew across the longitudinal study (kindergarten Cohen’s *d* = −1.42, eighth grade Cohen’s *d* = −3.38). The adolescents who participated in the current study were matched on chronological age. See Table 1 for participant characteristics.

**Table 1:** Participant characteristics

	Normal Language *n* = 18 (10 females)		SLI-Recovered *n* = 15 (7 females)		SLI-Persistent *n* = 19 (7 females)	
	Mean	SD	Mean	SD	Mean	SD
Chronological age	15.82	1.21	15.87	1.21	16.50	1.46
Cognitive standard score^a^	103.22	9.81	99.53	6.90	94.79	8.80
8th grade language composite *Z*-score	0.38	0.72	−0.47	0.38	−1.84	0.43
4th grade language composite *Z*-score	0.59	0.96	−0.62	0.47	−1.66	0.75
2nd grade language composite *Z*-score	0.52	0.95	−0.95	0.52	−1.85	0.48
Kindergarten language composite *Z*-score	0.67	0.98	−1.05	0.44	−1.69	0.46
Race	18 White		14 White 1 Black		16 White 3 Black	
Ethnicity	0 Hispanic		0 Hispanic		1 Hispanic	

*Note*. Chronological age at the time the ERP experiment was completed

^a^Performance IQ on the Wechsler Intelligence Scale for Children—Third Edition during the 8th grade visit. SLI classification for each visit followed the EpiSLI standard [74]

#### Ethics, consent, and permission

This study was approved by the institutional review board. All participants provided informed written assent or consent, and when necessary, parents or legal guardians provided informed written consent.

### Standardized assessments

Test batteries differed across the four visits in the longitudinal study in order to be developmentally appropriate. The eighth grade test battery included the Peabody Picture Vocabulary Test—Revised (PPVT-R; [11]) to assess receptive vocabulary knowledge and the Comprehensive Receptive and Expressive Vocabulary Test (CREVT; [78]) to assess expressive vocabulary. In addition, the Concepts and Following Directions and the Recalling Sentences subtests from the Clinical Evaluation of Language Fundamentals—Third Edition (CELF-3; [68]) were used to evaluate receptive and expressive grammatical skills. Lastly, the Qualitative Reading Inventory—Third Edition (QRI-3; [37]) tested narrative comprehension and production skills. Previous visits also included the Test of Language Development—Primary, 2nd edition (TOLD-P2; [47]) and a receptive and expressive narrative story task [9]. Scores from the battery of tests for each visit were used to create five composite scores, which were converted into *Z*-scores based on the entire dataset of the parent study [74]. Children were identified as having a language impairment if two or more language composite scores were 1.25 SD below the mean for the child’s chronological age group. The *Z*-scores were corrected to account for the disproportionate number of children with SLI in the sample relative to the prevalence in the general population. Additional details about diagnostic testing and the diagnostic EpiSLI standard is provided in Tomblin et al. [74].

Nonverbal intelligence was tested using the Performance Scale subtests in the Wechsler Intelligence Scale for Children—Third Edition (WISC-III; [81]). Handedness was measured by the Edinburgh Inventory for Assessment of Handedness [49]. All adolescents were right-handed, except for one adolescent in the SLI-Persistent group who was ambidextrous. Lastly, we confirmed that all adolescents had normal hearing with a hearing screening at a level of 20 dB HL at 500, 1000, and 2000 Hz, presented through headphones.

### Experimental task

Adolescents participated in a sentence judgment task that required them to judge whether or not each sentence was semantically and syntactically correct. Data from some of the current participants were presented in a previous study by Weber-Fox and colleagues [80]. The task contained 30 trials with a third-person singular subject and a verb correctly marked for agreement (i.e., third-person singular -*s*), 30 trials with a plural subject and a verb correctly marked for agreement, 30 trials with a third-person singular subject and a verb with an agreement omission error, 30 trials with a plural noun and a verb with an agreement commission error, 30 trials with a third-person singular subject and a semantically anomalous verb that is correctly marked for agreement, and 30 trials with a plural subject and a semantically anomalous verb that is correctly marked for agreement. A complete list of the stimuli appears in the Appendix. To enhance ecological validity, a female voice was recorded reading the stimuli sentences using normal prosody. The natural speech sentence stimuli were digitized at a rate of 16 kHz [79]. The auditory waveforms of the stimuli were visualized and the onset and offsets were identified using visual and auditory inspection to prevent clipping (using Cool Edit Pro software). The wave files of each word were saved as sound files. During the task, the sound file for each word was presented (using the Neuroscan STIM program) and followed by a 50-ms interstimulus interval. The sentences were approximately 3.5 s in duration (ranging from 2.7 to 4.9 s). The critical words (the verbs) were approximately 0.531 s in duration (ranging from 0.322 to 0.764 s). Codes for each word were inserted into the online EEG data recordings using Neuroscan, to allow for off-line data analysis of neural responses to the critical verbs of interest. The onsets of each word were clearly discernable, and the sentences maintained a natural-sounding rate, rhythm, and prosody.

The sentence stimuli were designed so that all of the words leading up to the critical word (the verb) were identical across the three conditions, which therefore allowed the ERPs elicited by the verbs in each condition to be directly compared. The sentences were presented in a counterbalanced manner in the following ways: (1) half of the noun subjects were singular and the other half were plural, and (2) each word that served as a control verb also served as a semantically anomalous verb in another sentence. The semantically anomalous verbs did not produce a frank anomaly in many cases, but were unexpected verbs given the preceding sentential context. This feature is an attribute of verbs given that verbs, unlike nouns, are quite flexible in use. Despite this, the anomalous verbs were apparent as the sentence continued (e.g., “Every day the ballerina submerges on her pointed toe shoes.”). The ERP components were measured at the point of the critical verb; therefore, the ERP data do not reflect the additional semantic information provided by the completion of the sentence. As such, the ERPs elicited by the verbs across the three conditions reflect processing of identical information leading up to the verb, and the additional information that follows the verb does not confound a comparison of the ERP measures across the three conditions.

#### Electroencephalographic recordings

**Fig. 1:**
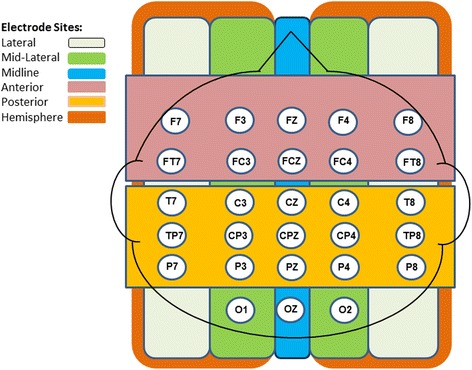
Head map. This figure depicts the organization of the EEG electrode sites

We measured electrical activity at the scalp using electrodes that were secured in an elastic cap (Quik-Cap, Compumedics Neuroscan). There were 28 electrodes (Ag-AgCl) that were positioned over homologous hemisphere locations according to the International 10-10 system [27]. Locations were as follows: lateral sites F7/F8, FT7/FT8, T7/T8, TP7/TP8, P7/P8; mid-lateral sites FP1/FP2, F3/F4, FC3/FC4, CP3/CP4, P3/P4, O1/O2; and midline sites FZ, FCZ, CZ, CPZ, PZ, OZ (see Fig. 1). Electrodes on the left and right mastoids served as the reference to the electrical recordings during data collection. Electrodes placed over the left and right outer canthi recorded horizontal eye movements. Vertical eye movement was monitored through recordings from electrodes placed over the left inferior and superior orbital ridges. We also adjusted all electrode impedances to 5 kΩ or less, amplified electrical signals within a bandpass of 0.1 and 100 Hz, and digitized online electrical signals (Neuroscan 4.0) at a rate of 500 Hz.

#### Procedures

After appropriate impedance levels were obtained, the participants sat in a sound-attenuating room and positioned 160 cm from a 47.5-cm monitor. The participants were instructed to listen to each sentence and then judge whether the sentence was semantically and syntactically correct. A fixation cross appeared on the screen, and, after a delay of 1000 ms, the sentence was presented binaurally through headphones at 70 to 75 dB SPL. The participants were asked to refrain from blinking during trials. After the presentation of each sentence, there was a 500-ms delay, followed by a “Yes/No?” prompt on the screen to cue the participant to press the “Yes” button if the sentence was semantically and syntactically correct or the “No” button if there was an error in the sentence. The response hands corresponding to the “Yes” and “No” buttons were counterbalanced across participants and sex. The sentence stimuli were presented in 5 blocks with 36 sentences in each block. Within each block, the sentences were pseudorandomized so that each condition was represented equally in each block.

#### ERP measures

The neural data were processed using EEGLAB, version 12-0-2-6b [10], and ERPLAB, version 5.0.0.0 [41, 40], which are MATLAB^©^ toolboxes (MathWorks, Natick, MA, USA). We used independent component analysis (ICA; EEGLAB [26]) to remove eye artifact. Specifically, ICA identifies independent sources of EEG signals. Components represent patterns from the EEG signal. Components that represent artifact, such as blinks, horizontal eye movements, and voltage drifts, were identified by two independent trained research assistants. Discrepancies were resolved by a third research assistant. Next, the EEG signals were low-pass filtered at 30 Hz with a 12-dB roll-off to remove high-frequency noise. After filtering, the data were epoched from 200 ms prior to the onset of the verb to 2500 ms post-stimulus to allow for averaging and ERP component measures. All of the EEG channels underwent automatic voltage-dependent thresholds to remove any trials that still contained artifact. The voltage-dependent thresholds were adjusted to take into account individual differences in EEG artifact amplitudes (e.g., size of blinks) to reliably reject true artifact without rejecting usable trials. The average voltage-dependent threshold for eye movement artifacts was 114 μV, and the average for the remaining artifacts (e.g., drift) at the remaining electrode sites was 209 μV. Each participant was required to contribute at least 20 artifact-free trials within each condition. The average number of usable trials in each sentence condition for each group was slightly higher than previous studies of auditory sentence processing in children and adolescents [21, 64]. Additionally, the number of artifact-free trials did not significantly differ across the groups, *F*(2, 49) = 3.07, *p* = .055. Finally, the EEG epochs of each individual were averaged for analysis of ERPs that were elicited by each task condition. Specifically, the ERP averages were triggered 200 ms prior to the verb onset in each sentence and included 2000 ms post-stimulus onset. The 200-ms interval preceding the onset of the critical verbs served as the baseline activity.

Adolescents’ brains are still undergoing neural maturation. Although the ERP components that were selected typically are most robust in the centroparietal regions, immature neural profiles often demonstrate a more distributed topography [24]. Therefore, of the 28 electrodes (Ag-AgCl) that were positioned over homologous hemisphere locations, we conducted omnibus analyses including the following electrodes: lateral sites F7/F8, FT7/FT8, T7/T8, TP7/TP8, P7/P8 and mid-lateral sites FP1/FP2, F3/F4, FC3/FC4, CP3/CP4, P3/P4.

Temporal windows for measuring the ERP mean amplitudes were selected after the grand averages were examined for each group. The windows were centered around the regions of maximal activity. In the current dataset, the same temporal windows were appropriate for each group’s grand averages. As a second step in the window selecting procedures, we examined each individual record to ensure the windows captured the components of interest (if present). The mean amplitudes of the N400 were measured within the temporal window of 370–570 ms, and the mean amplitudes of the P600 were measured within the temporal window of 800–1200 ms. The windows selected in these analyses are within the range of windows used to measure the N400 and P600 in children and adolescents and for stimuli that use connected speech (e.g., [46, 57, 65, 80]).

#### Analysis procedures

A repeated measures ANOVA tested whether behavioral performance differed according to condition (semantic vs. syntactic violations) and group and whether there was an interaction of condition and group. In order to control for response bias, *A*’ scores served as the dependent variable [19, 61]. Briefly, *A*’ scores serve as a measure of the proportion of correct responses in a two-alternative forced-choice task. The *A*’ value consists of scores from a control condition and an experimental condition (e.g., correct sentences and sentences with syntactic violations). The formula was *A*’ = 0.5 + (*y* − *x*) (1 + *y* − *x*) / 4*y* (1 − *x*), where *y* represents correct identifications (hits) and *x* represents incorrect identifications (false alarms; [38]). An *A*’ value of 1.00 represents perfect discrimination of correct and incorrect sentences. An *A*’ value of .50 indicates chance performance, for example, a “yes” response to 50% of the correct sentences and to 50% of the semantically anomalous sentences.

The ERP data were analyzed using a series of repeated measures ANOVAs. The omnibus models included condition (correct sentences vs. semantic/syntactic violation), hemisphere (right vs. left), laterality (lateral and mid-lateral), anterior-posterior (AP) order, and interactions across the predictor variables. When there was more than 1° of freedom in the numerator, the Huynh-Feldt (H-F) adjusted *p* value was used to determine significance [22].

In order to succinctly present the results, we report outcomes from the ERP measures recorded from the lateral and mid-lateral sites. Results from the midline electrode sites are also provided if they contribute additional information. Furthermore, given that the aim of the current study was to identify differences across language trajectory (i.e., group membership) and error type on behavioral and ERP data, we report significant results only for the group and error type factors and interactions between group and error type with each other, or other factors. Significant interactions among group, condition, and other predictor variables were followed up with step-down ANOVAs to better understand the significant effects.

Lastly, because high temporal resolution is a strength of ERPs, we conducted additional analyses that highlight time as a factor. To do so, we conducted sequential temporal analyses that examined three 50-ms temporal windows that lead up to and continued throughout the N400 and P600 time windows. Given that this approach increased the number of analyses that were conducted, we reduced the complexity of the statistical models by only examining the mean amplitudes of polarity shifts that came from electrodes in prespecified regions of interest. The regions of interest for the N400 effect were centroparietal electrode sites: C3, CZ, C4, CP3, CPZ, CP4, aligning with previous work examining the N400 [30] and visual inspection of the current data. The regions of interest for the P600 component were electrode sites: CP3, CPZ, CP4, P3, PZ, P4, aligning with previous work examining the P600 [18, 51] and visual inspection of the current data. The repeated measures ANOVAs contained the following factors: condition (correct vs. semantic/syntactic violation), group, electrodes, and interactions of group by condition, group by condition by electrodes, and condition by electrodes. In these analyses, we were most interested in whether there were interactions between group and condition, which had the potential to identify whether one group demonstrated earlier-onset ERP components elicited by the task stimuli. When appropriate, we conducted step-down analyses within each group to more accurately describe the nature of the group by condition interaction.

## Results

### Behavioral performance

**Fig. 2:**
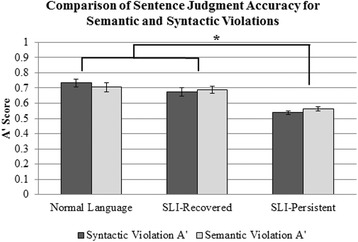
Comparison of *A*’ scores for syntactic and semantic violations across the groups. *Error bars* represent standard errors. Note: * *p* < .001

Behavioral performance across the conditions was examined first (see Table 2). When comparing *A*’ scores for the syntactic violation condition relative to the semantic violation condition, we found that there was no main effect of condition, *F*(1, 49) = 0.069, *p* = .793, indicating that performance was similar for the semantic violation and syntactic violation conditions. However, there was a main effect of group, *F*(2, 49) = 25.052, *p* < .001, *η*_p_^2^ = 0.506. Follow-up comparisons revealed that the NL and SLI-Recovered groups did not have significantly different *A*’ scores, *p* = .153, but the NL and SLI-Recovered groups had significantly higher *A*’ scores than the SLI-Persistent group, *p*s < .001. There was no interaction between condition and group, *F*(2, 49) = 1.209, *p* = .307 (see Fig. 2).

**Table 2:** Sentence judgment behavioral performance

	Normal Language		SLI-Recovered		SLI-Persistent	
	Mean	SD	Mean	SD	Mean	SD
Correct condition proportion correct	0.863	0.216	0.864	0.077	0.771	0.128
Semantic violation proportion correct	0.706	0.251	0.759	0.151	0.552	0.228
Syntactic violation proportion correct	0.781	0.195	0.715	0.244	0.443	0.168
Semantic violation *A*’	0.705	0.124	0.688	0.098	0.565	0.060
Syntactic violation *A*’	0.734	0.111	0.674	0.108	0.538	0.052
Omission violation *A*’	0.744	0.121	0.681	0.108	0.555	0.067
Commission violation *A*’	0.726	0.109	0.673	0.119	0.528	0.046

*Note. A*’ scores of .5 indicate chance performance

**Fig. 3:**
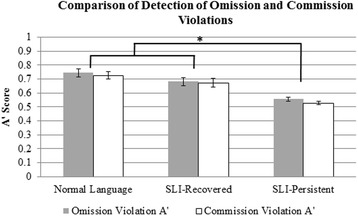
Comparison of *A*’ scores for omission violations and commission violations across the groups. *Error bars* represent standard errors. Note: *p < .001

Next, we compared behavioral performance on the omission and commission trials. A repeated measures ANOVA tested whether there was a difference between omission and commission *A*’ scores, a difference between groups, and whether there was an interaction of error type and group. The difference between error type did not reach significance, *F*(1, 49) = 3.879, *p* = .055, *η*_p_^2^ = 0.073; omission *A*’ scores were only slightly higher than commission *A*’ scores. There was a significant effect of group, *F*(2, 49) = 21.399, *p* < .001, *η*_p_^2^ = 0.466. Post hoc comparisons revealed that the NL and SLI-Recovered groups had significantly higher *A*’ scores than the SLI-Persistent group, *p*s < .001, but differences between the NL and SLI-Recovered groups did not reach significance, *p* = .079. Lastly, there was no interaction of error type and group, *F*(2, 49) = 0.356, *p* = .702 (see Fig. 3).

### ERP patterns elicited by sentence processing

Our second research question asked whether ERPs elicited from the sentence processing task differentiated the three groups. Below, we present our findings for each violation type.

#### Semantic violation N400 results

**Fig. 4:**
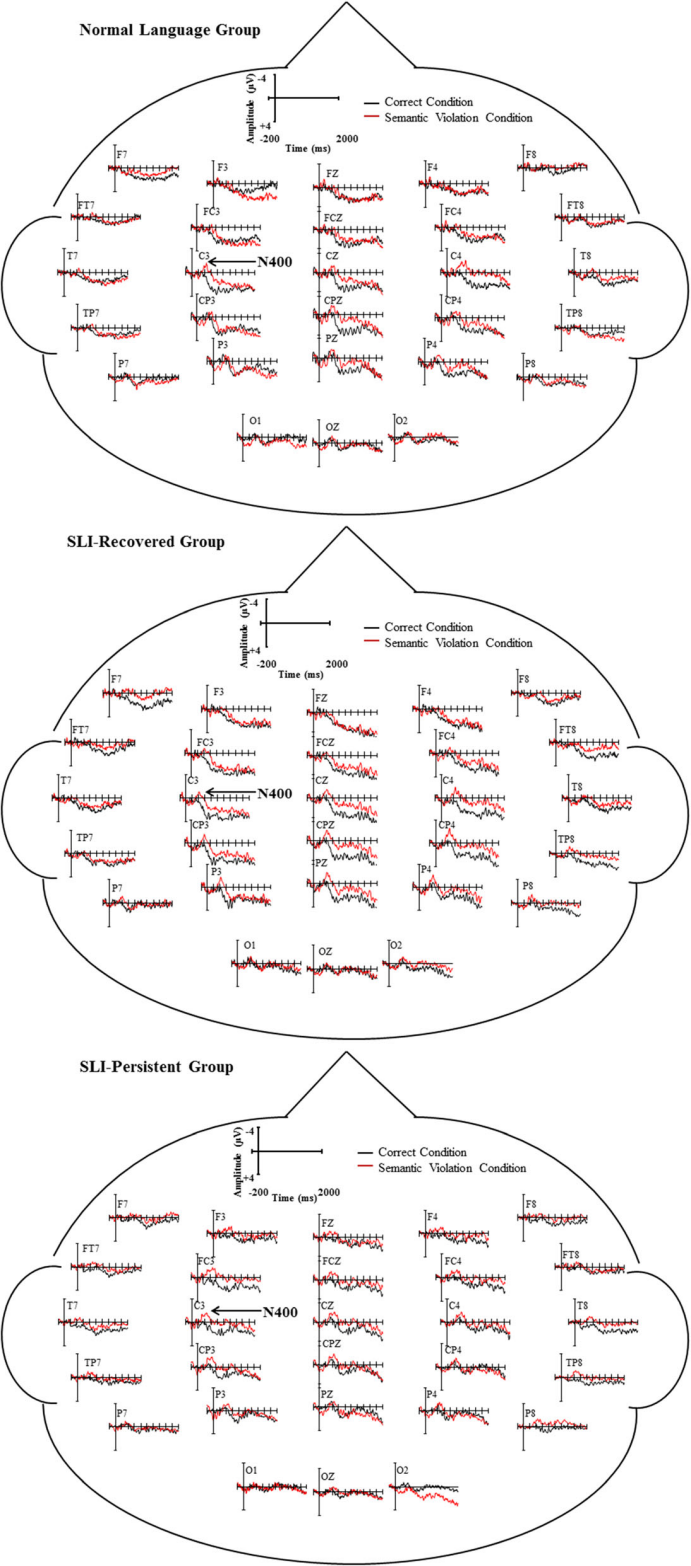
Head maps by group for correct and semantic violation conditions

The mean amplitude in the N400 window (370–570 ms following the onset of the verb) was analyzed to examine lexical processing during correct sentences and sentences with semantic errors. There was a significant effect of condition, *F*(1, 49) = 11.318, *p* < .001, *η*_p_^2^ = 0.188, with an N400 effect appearing in the semantic violation condition. There was no group or group by condition effect, *p*s > .35. However, there was a significant interaction between condition and laterality, *F*(1, 49) = 44.099, *p* < .001, *η*_p_^2^ = 0.241, identifying a larger amplitude distribution over mid-lateral compared to lateral electrode sites. Additionally, there was a significant interaction across condition, hemisphere, laterality, and group, *F*(2, 49) = 3.613, *p* = .003, *η*_p_^2^ = 0.210. Figure 4 depicts the ERPs elicited by verbs in the correct sentences and sentences with a semantic violation for each group.

##### N400 sequential temporal analysis

In order to examine additional information about the timing of the emergence of the N400 across the groups, we conducted a series of sequential temporal analyses on 50-ms temporal windows leading up to and comprising the N400 time window. Within our region of interest (C3, CZ, C4, CP3, CPZ, CP4), there were no main effects or interactions in the three 50-ms temporal windows preceding the N400 time window. During the 50-ms temporal windows that made up the N400 time window, each of the analyses yielded a main effect of condition, indexing the negative polarity shift that was elicited by the semantically anomalous verb. However, there was not a significant interaction of group by condition or group by condition by electrodes (*p*s > .45). There also were no group differences (*p*s > .20), but there was a main effect for electrodes (*p*s < .02), indicating that the N400 component was left-lateralized.

#### Omission violation P600 results

**Fig. 5:**
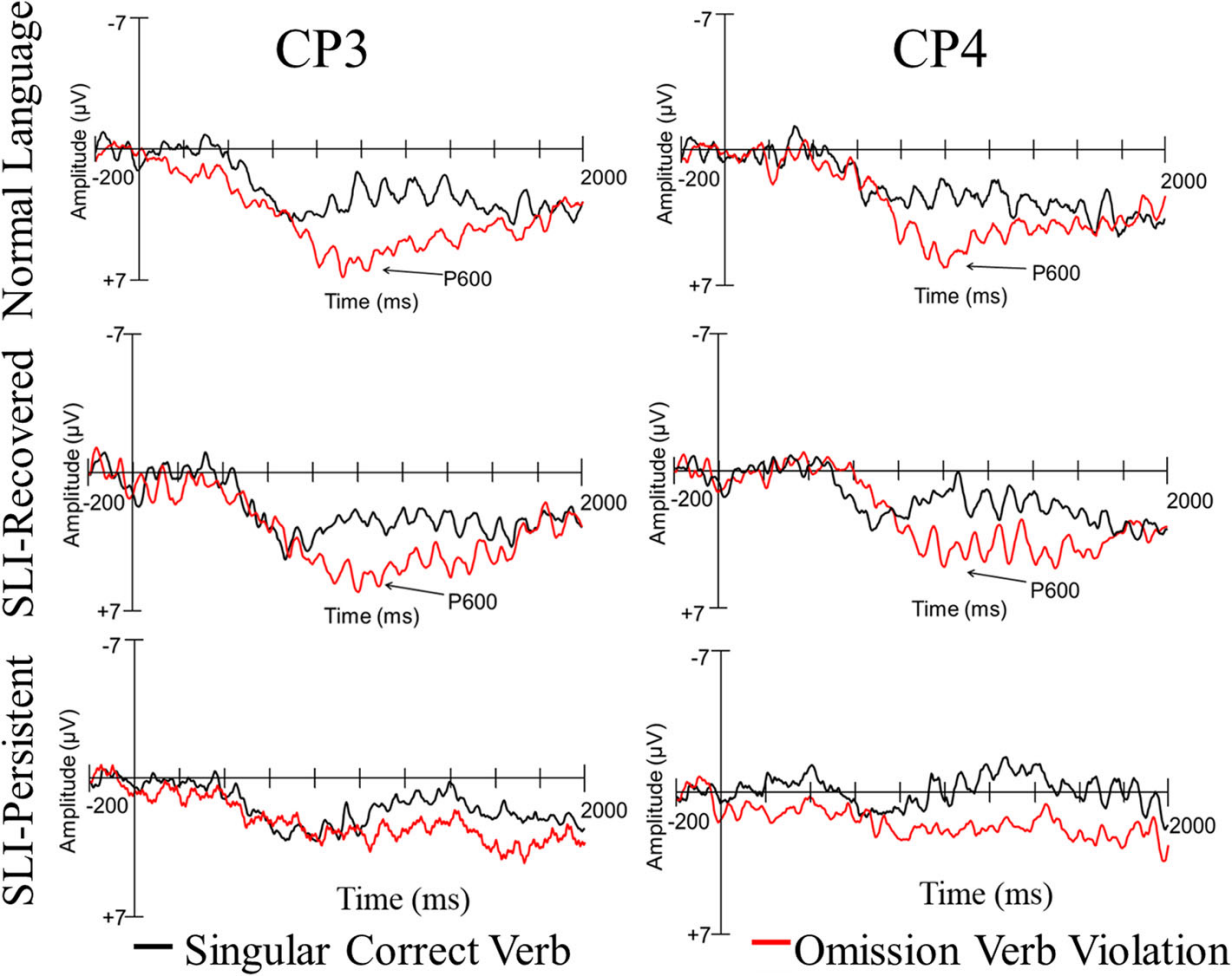
Omission violation ERP group patterns

The mean amplitude in the P600 window (800–1200 ms following the onset of the verb) was examined to test the singular sentences with the third-person singular -*s* correctly marked and the sentences with omission violations. As depicted in Fig. 5, there was a larger P600 component elicited by omission violations compared to correctly marked verbs, *F*(1, 49) = 11.596, *p* < .001, *η*_p_^2^ = 0.191. There was no group effect, *p* = .442; however, there was a marginal interaction between condition and group, *F*(2, 49) = 2.715, *p* = .076, *η*_p_^2^ = 0.100. In addition, there was a significant interaction between condition and laterality, *F*(1, 49) = 14.626, *p* < .001, *η*_p_^2^ = 0.230, indicating that there was larger amplitude distribution in the mid-lateral electrode sites for the omission condition. There also was a significant interaction between condition and AP, *F*(4, 196) = 3.345, *p* = .011, *η*_p_^2^ = 0.064, with greater positivity in the posterior electrode sites during the omission condition. A three-way interaction across condition, AP, and hemisphere, *F*(4, 196) = 3.425, *p* = .019, *η*_p_^2^ = 0.065, indicated that greater positivity appeared during the omission condition in the posterior electrodes in the left hemisphere. Lastly, there was a significant interaction between group and laterality, *F*(2, 49) = 6.072, *p* = .004, *η*_p_^2^ = 0.199, indicating that there was larger amplitude distribution in the mid-lateral electrode sites than the lateral electrode sites for the NL and SLI-Recovered groups, relative to the SLI-Persistent group.

Because the marginal interaction between group and condition may provide further insight into the neural processing of omission violation errors, we conducted a series of step-down repeated measures ANOVAs within each group. The ANOVA in the NL group revealed a significant effect of condition, *F*(1, 17) = 8.566, *p* = .009, *η*_p_^2^ = 0.335. There also was a significant interaction of condition and laterality, indicating a stronger positivity elicited by the omission condition on the mid-lateral electrode sites, *F*(1, 17) = 7.039, *p* = .017, *η*_p_^2^ = 0.293. The SLI-Recovered group also demonstrated a significant effect of condition, *F*(1, 14) = 8.798, *p* = .010, *η*_p_^2^ = 0.386. Conversely, the SLI-Persistent group did not demonstrate a condition effect, *F*(1, 18) = 0.013, *p* = .911, *η*_p_^2^ = 0.001. However, there was a significant three-way interaction of condition, hemisphere, and AP, *F*(4, 72) = 2.935, *p* = .048, *η*_p_^2^ = 0.140, indicating that there was greater positivity in the omission condition relative to the correct condition in the posterior electrode sites in the left hemisphere. The SLI-Persistent group demonstrated a marginally more restricted P600, relative to the NL and SLI-Recovered groups.

##### P600 omission violation sequential temporal analysis

Next, we conducted a series of sequential temporal analyses on 50-ms temporal windows leading up to and comprising the P600 time window. Within our region of interest (CP3, CPZ, CP4, P3, PZ, P4), there were no main effects or interactions of group, condition, or group by condition in the three 50-ms temporal windows preceding the P600 time window. When examining each 50-ms time bin within the P600 time window, we found that the 800- to 850-ms temporal window yielded a significant effect of condition (*p* = .033) and electrodes (*p* = .033) and, importantly, a significant interaction across group, condition, and electrodes (*p* = .043). There was no main effect of group (*p* = .059), and no interaction of group by condition or electrodes by group (*p*s > .65). Within-group analyses were conducted to more accurately describe the interaction across group, condition, and electrodes. None of the within-group analyses yielded a main effect of condition (*p*s > .10); however, the SLI-Recovered group had a significant interaction between condition and electrodes (*p* = .041). This indicates that the SLI-Recovered group had an earlier neural response in the medial and left electrode sites that differentiated the correct verbs and the verbs with omission violations.

In the remaining 50-ms temporal windows (ranging from 850 to 1200 ms), there was a consistent effect of condition (*p*s < .003), indicating that the omission violations elicited a P600, and an effect of electrodes (*p*s < .001), indicating that larger amplitudes were observed in the left and medial electrode sites. In addition, the 900- to 950-ms temporal window analysis yielded a significant effect of group (*p* = .003), with the NL and SLI-Recovered groups having significantly higher positivity relative to the SLI-Persistent group. The 50-ms temporal window analyses starting at 950, 1050, and 1150 ms also yielded significant effects of group (*p*s < .05), with the NL group demonstrating significantly larger amplitude P600 relative to the SLI-Persistent group.

#### Commission violation P600 results

Following our analyses of the omission violations, we examined the mean amplitude in the P600 window (800–1200 ms following the onset of the verb) for the plural sentences with correct verb agreement and plural sentences with commission violations. Unlike the omission condition, there was no main effect of the commission violation condition, relative to the correct sentences with plural subjects, *p* = .629. However, there was a significant three-way interaction of condition, AP, and laterality, *F*(4, 196) = 2.592, *p* = .049, *η*_p_^2^ = 0.050, indicating that there was greater positivity in the posterior mid-lateral electrodes in the commission condition. Moreover, there was a significant effect of group, *F*(2, 49) = 4.952, *p* = .011, *η*_p_^2^ = 0.168, with the NL and SLI-Recovered groups having overall significantly greater positivity than the SLI-Persistent group (*p*s < .03), but similar positivity in the NL and SLI-Recovered groups (*p* = .561). Lastly, there was not a significant interaction between group and condition, *p* = .180.

**Fig. 6:**
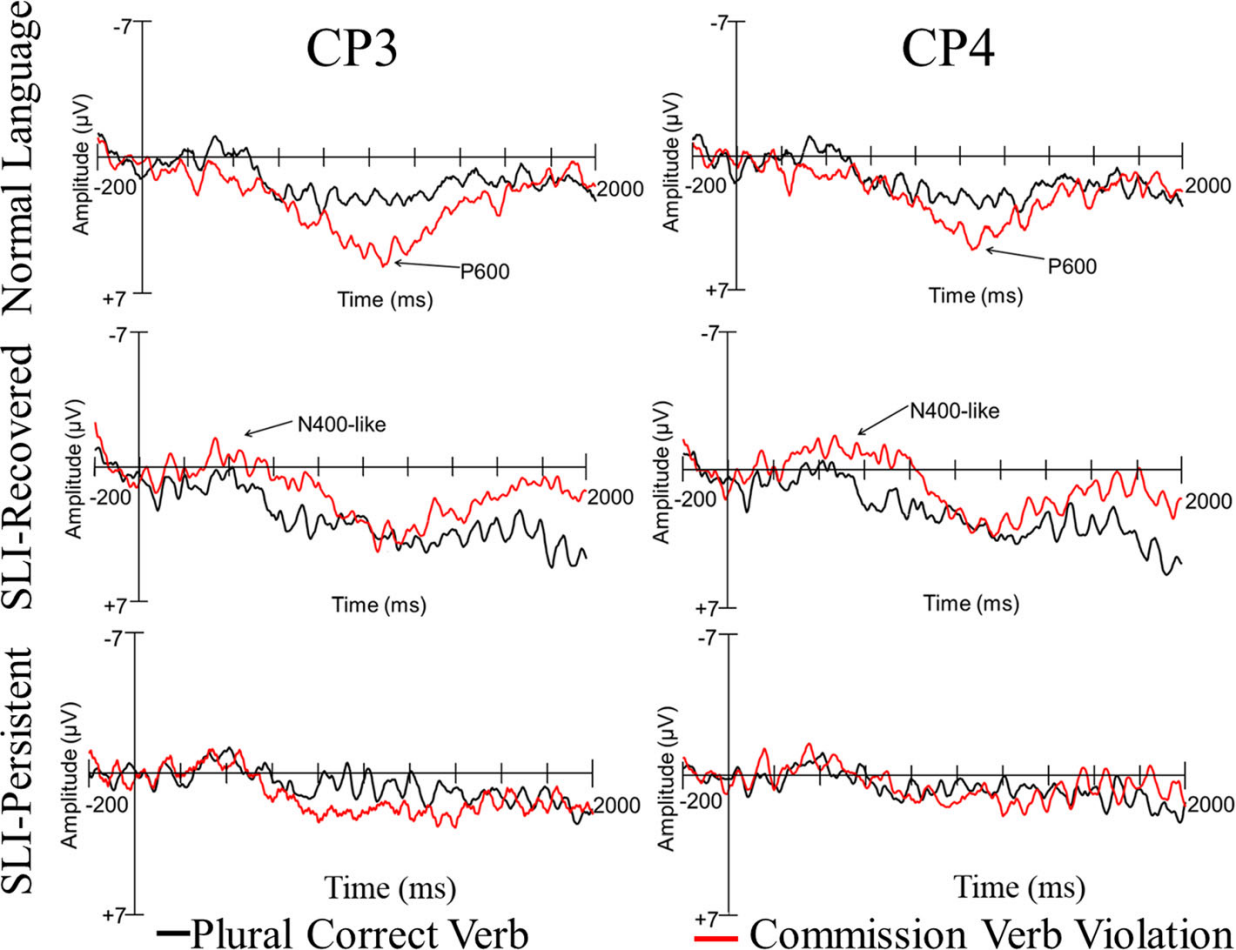
Commission violation ERP group patterns

After visualizing the waveforms in the plural control and commission conditions (see Fig. 6), it was apparent that each group presented with a unique pattern of neural activity. Specifically, children in the NL group demonstrated a robust P600 in the commission condition at electrode sites over more medial and posterior brain regions. In contrast, the SLI-Recovered group demonstrated a differing profile with an earlier N400-like negativity that was followed by a positive-moving polarity shift, which did not exceed the positivity that was also elicited in the correct plural sentences. Although the positivity observed in the commission agreement errors and correct plural sentences was mostly overlapping, there was a greater overall change in polarity elicited by the commission agreement errors given the morphology of the ERP waveforms. Lastly, the SLI-Persistent group portrayed a restricted P600 amplitude over the left centroparietal sites for the commission violation condition.

##### Negativity related to commission violations

To further examine the negativity in the SLI-Recovered group, we conducted a repeated measures ANOVA on the mean amplitude of the waves between 600 and 800 ms after the onset of the verb. This temporal window was centered around the negativity elicited in the SLI-Recovered group. There was no significant effect of condition; however, there was an interaction between group and condition, *F*(2, 49) = 4.452, *p* = .017, *η*_p_^2^ = 0.154. Therefore, we conducted step-down ANOVAs within each group to examine the patterns associated with the commission violations. There were no significant findings in the NL group analyses. Within the SLI-Recovered group analysis, there was a main effect of condition, *F*(1, 14) = 11.516, *p* = .004, *η*_p_^2^ = 0.451, with larger amplitude negativity elicited by the commission violation relative to the correct condition. Lastly, the SLI-Persistent group analyses revealed a marginal interaction across condition, hemisphere, and AP, *F*(4, 72) = 2.480, *p* = .078, *η*_p_^2^ = 0.121, with greater amplitude positivity observed in the posterior electrodes in the left hemisphere during the commission condition. There also was a marginal interaction across condition, AP, and laterality, *F*(4, 72) = 2.098, *p* = .096, *η*_p_^2^ = 0.104, indicating that there was slightly greater amplitude positivity distributed over the mid-lateral posterior electrode sites.

##### Sequential temporal analyses for the commission violation

**Fig. 7:**
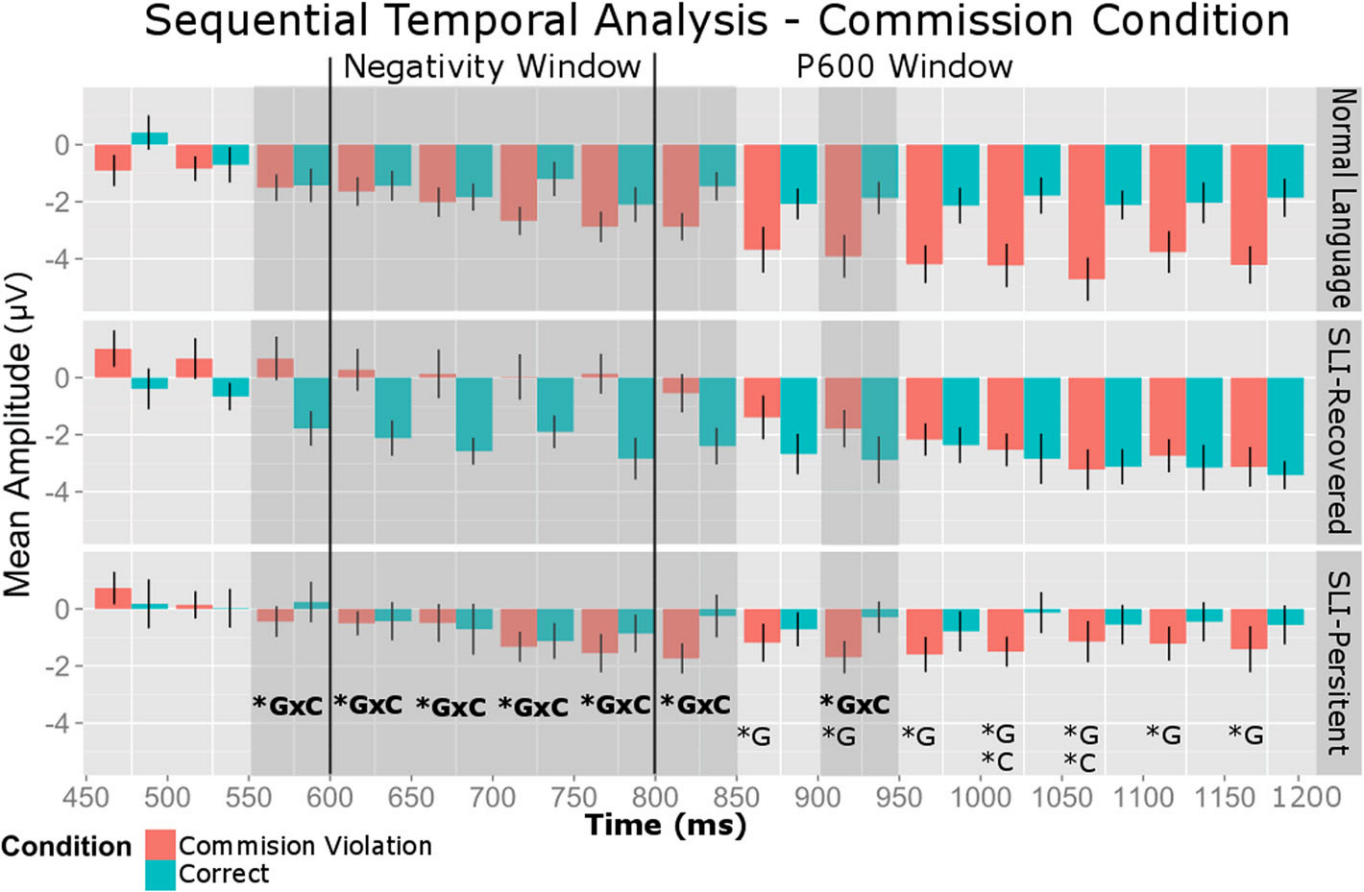
*p < .05 for the repeated measures ANOVAs that included condition, electrodes, group, and interactions across condition, electrodes, and group. All repeated measures ANOVAs had significant main effects of electrodes. *G* main effect of group, *C* main effect of condition, *GxC* group by condition interaction. At 850–900, 900–950, and 950–1000 ms, the NL group had a larger positive amplitude relative to the SLI-Persistent group. At 1000-1050, 1050–1100, 1100–1150, and 1150–1200 ms, the mean amplitude across the conditions in the NL and SLI-Recovered groups was larger than that in the SLI-Persistent group, supporting the omnibus P600 finding that the NL and SLI-Recovered groups had overall significantly greater positivity than the SLI-Persistent group. The interactions between group and condition highlighted the different profiles in the negativity window, contributing to the SLI-Recovered group’s unique neural profile. The NL group had an earlier-emerging P600 component elicited by the commission violation relative to the other groups (highlighted in the 900–950-ms temporal window)

As in the previously discussed conditions, we conducted a series of sequential temporal analyses on 50-ms temporal windows that preceded the negativity window and 50-ms temporal windows that captured the negativity and P600 time windows. Our region of interest included electrode sites CP3, CPZ, CP4, P3, PZ, and P4. Our results are presented in Fig. 7. Briefly, the sequential temporal analyses revealed several group by condition interactions. Most of the findings highlighted the different profiles in the negativity window, highlighting the SLI-Recovered group’s unique neural profile. Our findings also revealed a significant interaction of group by condition in the 900- to 950-ms temporal window. The follow-up analyses conducted within the groups revealed that there was a significant condition effect in the NL group, but not the SLI-Recovered or SLI-Persistent groups, indicating that the NL group may have had an earlier-emerging P600 component that was elicited by the commission violation condition relative to the other groups.

#### Exploration of individual differences

**Fig. 8:**
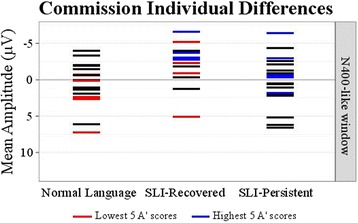
Commission individual differences. This scatterplot depicts the individual differences in mean amplitude differences (sentences with commission violations minus sentences with correct verbs with plural subjects) for our regions of interest (CP3, CPZ, CP4, P3, PZ, P4) for the negativity time window. *Blue lines* highlight five participants within the SLI-Recovered and SLI-Persistent groups who had the highest behavioral accuracy scores (*A*’). The *red lines* depict the five lowest scoring adolescents in the Normal Language and SLI-Recovered groups. See text (individual differences) for the corresponding ranges of *A*’ scores

Lastly, we explored the individual differences within our participants. Figure 8 depicts the individual variability of the neural correlates of processing sentences with commission violations, relative to the sentences with correct verbs and plural subjects, within and across the groups. Specifically, we explored the relationship between the mean amplitude of the negativity window and the behavioral *A*’ accuracy scores. As such, Fig. 8 depicts five adolescents in the Normal Language and SLI-Recovered groups who had the lowest *A*’ scores for the commission condition (depicted in red; Normal Language range .499–.668, SLI-Recovered range .502–.602) and five adolescents in the SLI-Recovered and SLI-Persistent groups with the highest behavioral performance (depicted in blue; SLI-Recovered range .830–.800, SLI-Persistent range .683–.541). Furthermore, to provide insight into whether the early negativity may have indicated a compensatory route to support performance on our language processing task in our adolescents with a history of SLI and persistent SLI, we conducted a bivariate correlation analysis. We tested for a correlation between the *A*’ accuracy scores for the commission violation condition and mean amplitude differences in the negativity window for our region of interest (CP3, CPZ, CP4, P3, PZ, P4). There was a significant relationship between our variables of interest, *r* = −0.438, *p* = .010, such that the higher *A*’ scores were associated with greater mean amplitudes in the negativity window, when presented with a commission violation sentence.

## Discussion

The current study examined language processing at the behavioral and neural levels in adolescents who followed different language trajectories. Our results indicate that the three groups demonstrated different language processing profiles. Most notably, we found that although adolescents in the SLI-Recovered group had similar behavioral performance to their typically developing peers, they appear to have processed some aspects of the language stimuli in a different manner. We discuss our specific findings below.

### Sentence processing behavioral accuracy

The sentence processing task yielded a rich dataset that tested both semantic and syntactic processing of verbs. Although it is informative to examine both semantic and syntactic features of verb processing, including both types of judgments (semantic and syntactic accuracy) in the same task likely placed high metalinguistic demands and contributed to the relatively lower *A*’ scores for all of the groups compared to scores seen in previous studies with semantic violations [46, 64]. It is also likely that the reduced accuracy in identifying semantic violations was due to the inclusion of verbs with low cloze probability. Nevertheless, the NL and SLI-Recovered groups had *A*’ scores that were well above chance.

Not surprisingly, accuracy for the SLI-Persistent group was considerably lower (mean *A*’ = .575). The reduced accuracy on the semantic violation trials, in addition to syntactic violation trials, supports previous findings that suggest that children with SLI have weak semantic representations [1, 65, 70]. Furthermore, poor performance in identifying anomalous verbs provides additional support that children with SLI have difficulties processing verbs [2, 4, 32]. The lexical-semantic difficulties in the SLI-Persistent group may have become more apparent in the current study given the coupling of the additional metalinguistic task demands and nonverbal processing difficulties that many children with SLI experience [32, 35]. In fact, the dual monitoring nature of the task likely increased the processing demands that are associated with executive function abilities. Given that children with SLI have been found to have weaknesses in executive function abilities [43, 44, 82] and that executive function abilities have been found to be associated with lexical processing [20], the task design may have exacerbated lexical-semantic processing weaknesses in the SLI-Persistent group.

#### Behavioral processing of omission and commission errors

Although children typically produce omission and not commission errors in expressive language, contrary to our predictions, commission violations were not more easily detected relative to omission errors. In fact, although our results only approached significance, adolescents in our sample had slightly higher accuracy in the omission condition. Rice and colleagues [61] found that children with and without SLI correctly reject commission errors, paralleling their productive speech. However, Rice and colleagues tested commission errors that differed from ours, including copula and auxiliary *be* and third-person singular -*s* in combination with first-person singular subjects (e.g., “He *are* sad.”, “I *likes* cake.”). It is possible that some types of agreement errors may have disproportionally influenced child accuracy. It is unclear whether Rice and colleagues would have observed similar results if they tested the particular commission errors used within the present study.

In contrast to the Rice et al. [61] study, Redmond and Rice [58] focused on one category of finiteness markers, the irregular past tense. Redmond and Rice found that 8-year-old children with SLI or a history of SLI were more likely than age-matched peers to accept omission and commission errors, even when their productive language did not include commission errors. Although not directly tested, children with SLI had lower accuracy in detecting omission errors than commission errors; age-mates correctly identified both errors with similarly high accuracy [58]. Miller and colleagues [45] also found that identifying past tense -*ed* omission errors was particularly challenging in adolescents with typical language and language impairment. However, the children performed equally well at identifying commission and omission errors of the third-person singular -*s*.

Lastly, Leonard and colleagues [36] found that, in a less metalinguistically demanding word detection task, adolescents with SLI, nonspecific language impairment (NLI), and typical development were slower to respond to target words during the commission condition, relative to the grammatical condition. Reduced sensitivity to finiteness marking errors was only observed in the SLI and NLI groups during the omission condition. However, the omission and commission conditions contained errors of past tense -*ed* and third-person singular -*s*. Leonard et al. [36] also found that adolescents responded more quickly during past tense -*ed* items than third-person singular -*s* items, but could only speculate as to why this was the case. Therefore, processing of omission and commission errors may differ according to the specific grammatical marker.

### Neural profiles of language processing

#### Semantic violation processing

Despite the below-ceiling behavioral accuracy on judging correct sentences and sentences with an anomalous verb, all groups displayed an N400 in the semantic violation condition and there were no differences in the onset of the N400 across the groups, as indicated by our sequential temporal analysis of the N400. As previously noted, the anomalous verbs used in the current study were not frank violations, which may have allowed for the open-class anomalous verbs to be more easily integrated into the sentences than frank violations of high cloze class probability words. Previous studies have used more frank semantic violations and therefore demonstrated more robust N400s than the current study. This example with open-class semantic verb violations may demonstrate adolescents’ abilities to gather the gestalt of the sentence despite the presence of subtle infelicities [14, 71].

Regardless, our findings are in line with results from a subset of these data presented by Weber-Fox et al. [80] and extend it to including information on lexical integration in adolescents with a history of SLI (i.e., SLI-Recovered group). In addition, our results mirror findings presented by Fonteneau and van der Lely [15] in suggesting that, at least in older children and adolescents with SLI, neural profiles of lexical integration do not seem to robustly differ from typically developing peers (but see studies of lexical integration in earlier developmental periods, e.g., [17, 64]).

Although we did not observe a significant group effect or interaction of group and condition, there was a significant interaction across condition, hemisphere, laterality, and group. Complex interactions like these can be difficult to interpret without numerous post hoc analyses. However, one potential implication of this interaction is that the SLI-Persistent group had a broader distribution of the N400 component, relative to the NL and SLI-Recovered groups. The broader distribution of the N400 component resembles a less mature neural profile of lexical integration [3, 25]. However, additional work is needed to explore potential differences in neuromaturational profiles in children with typical and atypical language development. In addition, it is possible that adolescents in the SLI-Recovered group were able to overcome early neuromaturational delays, whereas the adolescents in the SLI-Persistent group experienced a persistent lag in neuromaturation [39]. Therefore, slight variations in semantic processing at the neural level may persist in adolescents across a continuum of language abilities [56]. This interaction, combined with the differences in behavioral performance, suggests that semantic processing of verbs within sentences may still pose slight challenges for some adolescents with SLI or a history of SLI.

#### Omission violations

The typical ERP profile of syntactic violations, with a P600 component, was observed for the NL and SLI-Recovered groups. Despite early language impairments, adolescents in the SLI-Recovered group demonstrated similar behavioral performance and neural profiles to adolescents with no history of language impairments. Although differences may have been detected at earlier points in development, the adolescents in the SLI-Recovered group may have made sufficient language proficiency gains and maturational changes to demonstrate a more typical neural profile while processing sentences with omission errors.

In contrast, there was a marginal difference in the P600 component elicited from omission violations in adolescents in the SLI-Persistent group. They demonstrated a marginally more restricted distribution of the P600 in the posterior region of the left hemisphere, as evidenced by the interaction across condition, hemisphere, and AP. Previous work designed to use the P600 component to examine the processes related to verb agreement errors in children with SLI is lacking. In one study using an overlapping dataset with the current study, Weber-Fox and colleagues [80] also found that adolescents with SLI did not demonstrate a P600 component after listening to verb agreement violations of the third-person singular -*s* marker, but typically developing adolescents did. These results differ from studies examining other types of syntactic errors, such as wh- dependencies and word category violations [15, 65]. Although adolescents with persistent language impairment no longer produce consistent omission errors in their expressive language, difficulties in processing verb agreement omission errors may persist at both the behavioral and neural levels.

#### Commission violations

The current study extended previous work by separately examining omission and commission errors of the third-person singular -*s* tense marker. We found that, when examining plural correct sentences and sentences with commission errors, the adolescents in the NL and SLI-Recovered groups demonstrated overall significantly greater positivity in the P600 window than the adolescents in the SLI-Persistent group. In addition, the sequential temporal analyses indicated that the NL group demonstrated an earlier onset of the P600 component relative to the other groups. Purdy and colleagues [57] observed a P600 component after hearing a local verb agreement commission error and a negative polarity shift in the anterior regions of the brain (i.e., anterior negativity) in both children with typical development and children with a history of SLI. In the current study, visual inspection of the group waveforms revealed that the adolescents in the SLI-Recovered group also demonstrated a negative polarity shift after a commission error, which was followed by similar positive mean amplitudes for the plural correct sentences and the sentences with commission errors. The negativity observed in the current SLI-Recovered group, however, was observed more globally and temporally later, causing it to be more characteristic of an N400 than the AN component.

The unique neural profile, indexed by the N400-like negativity, observed in the SLI-Recovered group was one of the most important and novel findings in the current study. Although syntactic deficits are more often associated with the AN component, later-occurring negativities resembling the N400 component have been found for morphosyntactic violations (e.g., [48, 69]). As such, recent work has suggested that the dominance of the N400 or the P600 component provides insight into the competition between two interactive processing “streams” [29, 52, 77]. One stream is associated with the N400, indexing lexically or memory-based processing, and the other is associated with the P600 and thought to indicate combinatorial, algorithmic processing that often relies on linguistic constraints such as morphosyntactic rules [29, 30, 52]. Furthermore, it is thought that the robustness of the N400 and P600 may provide insight into the processing stream that is engaged during language processing.

Along this line, recent studies have begun to explore individual differences in relation to neural profiles during language processing. For example, Pakulak and Neville [53] observed that monolingual adults with high language proficiency demonstrated the well-documented AN and P600 waveforms following phrase structure violations. In contrast, adults with relatively low language proficiency demonstrated a later negativity waveform that was N400-like, in addition to a smaller P600 component. Additional work has documented individual differences in neural profiles that appeared to be associated with language proficiency [73] and chronological age [67]. These findings, along with our findings, suggest that there are several neurocognitive routes to grammatical comprehension.

Our sample of adolescents in the SLI-Recovered group resembled the NL group in their behavioral accuracy in the language processing task; it is possible that these individuals developed compensatory routes to successfully process language despite early language weaknesses. In fact, the Procedural Deficit Hypothesis predicts that individuals with SLI may rely on declarative memory to compensate for procedural memory weaknesses [76]. Behavioral work also has demonstrated that declarative memory predicts syntactic abilities in children with SLI [8]. In the current study, across adolescents in the SLI-Recovered and SLI-Persistent groups, we found a significant correlation between the *A*’ accuracy scores for the commission violation condition and mean amplitude differences in the negativity window. An alternative explanation may be that the N400-like pattern observed in the SLI-Recovered group may have emerged because the task required monitoring of both semantic and syntactic aspects of sentences; however, it is unclear why the task demands would elicit this robust neural profile for only the SLI-Recovered group. Additional work examining individual differences that measure behavioral and neural indices of language processing is needed.

### Limitations

Despite the unique insights the current study provides, there are limitations that we would like to address. First, we were unable to limit our analyses to examining correct trials only. We required the adolescents to contribute at least 20 artifact-free trials within each condition. Given the challenging nature of the task and given that we were examining language processing in adolescents who have language impairments, we would have had to drastically reduce the number of participants in each group if we had required 20 artifact-free correct trials. Despite this limitation, we explored the waveforms for the correct trials only for each group and found that the neural profiles were similar to the waveforms that included all trials regardless of accuracy. This leads us to believe that we would have found similar results had we been able to analyze correct trials only. Relatedly, although our task yielded a rich dataset, it would have been more ideal to have been able to have an equal number of (semantically or syntactically) correct and incorrect sentences. To do so, we would have had to increase the number of trials in the task, which may have fatigued our participants and increased data loss associated with fatigue.

## Conclusions

The current study expanded on previous studies to contribute needed information about the trajectory of language impairments. We observed that adolescents in the SLI-Persistent group had lower accuracy on the sentence judgment task than the NL and SLI-Recovered groups. In addition, the SLI-Persistent group demonstrated a restricted P600 after listening to syntactic violations. Furthermore, although sentence processing accuracy was slightly lower in the SLI-Recovered group relative to the NL group, differences did not reach significance. This suggests that adolescents who had a history of SLI but later scored in the normal range of language assessments may have experienced sufficient language gains through therapy and/or development to enhance language processing abilities. Although subtle weaknesses may still be present, on the whole, they did not appear to fit under an “illusory recovery” classification. More interesting, though, is the way in which adolescents in the SLI-Recovered group processed language. The ERP data from the commission condition indicated that ERPs from adolescents in the SLI-Recovered group may have reflected compensatory strategies to facilitate some aspects of language processing during the task. Specifically, they appear to have recruited lexical processing streams to support syntactic processing. Additional work examining underlying neural indices of language processing in children with a history of SLI is greatly needed. Furthermore, future work should strive to collect neural data longitudinally to contribute much needed information about the trajectory of language impairments and the neural mechanisms that underlie developmental changes.

## Appendix

### Correct verb (verb agreement violation, semantic violation)

1. Every day, the horses *gallop* (*gallops, sing*) to the top of the hill.

2. Every day, the canaries *sing* (*sings, gallop*) at the top of their lungs.

3. Every day, the cow *grazes* (*graze, types*) to the top of the hill.

4. Every day, the secretary *types* (*type, grazes*) many legal documents.

5. Every day, the ballerina *dances* (*dance, submerges*) on her pointed toe shoes.

6. Every day, the submarine *submerges* (*submerge, dances*) to a depth of five hundred feet.

7. Every day, the beautician *style* (*styles, slither*) at least 30 heads.

8. Every day, the snakes *slither* (*slithers, style*) through the fallen leaves.

9. Every day, the lieutenant *salutes* (*salute, meows*) his captain during routine inspections.

10. Every day, the cat *meows* (*meow, salutes*) when he thinks he is going to be fed.

11. Every day, the grandmothers *knit* (*knits, peck*) scarves and sweaters for their friends and families.

12. Every day, the chickens *peck* (peck*s, knit*) at the dirt for bits of dried corn.

13. Every day, the earrings *dangle* (*dangles, cry*) from her earlobes.

14. Every day, the babies *cry* (*cries, dangle*) when they are hungry.

15. Every day, the customers *gossip* (*gossips, swoop*) about the hot news in town.

16. Every day, the eagles *swoop* (*swoops, gossip*) down to hunt for their food.

17. Every day, the mailman *delivers* (*deliver, growls*) our letters and packages with care.

18. Every day, the dog *growls* (*growl, delivers*) when someone passes his yard.

19. Every day, the teachers *assign* (*assigns, gnaw*) on the old boxes stored there.

20. Every day, the mice *gnaw* (*gnaws, assign*) on the old boxes stored there.

21. Every day, the children *pretend* (*pretends, rust*) to be superheroes.

22. Every day, the cars *rust* (*rusts, pretend*) a little bit more.

23. Every day, the senator *votes* (*vote, perches*) on important issues.

24. Every day, the bird *perches* (*perch, votes*) at the top of our tree.

25. Every day, the owner *rents* (*rent, backfires*) his rooms for reasonable rates.

26. Every day, the engine *backfires* (*backfire, rents*) on my way to work.

27. Every day, the farms *plow* (*plows, buzz*) their corn and soybean fields.

28. Every day, the bees *buzz* (*buzzes, plow*) happily as they make honey.

29. Every day, the president *plans* (*plan, blasts*) his next course of action.

30. Every day, the wind *blasts* (*blast, plans*) the lakefront residents.

31. Every day, the officers *arrest* (*arrests, shimmer*) people who break the law.

32. Every day, the pennies *shimmer* (*shimmers, arrest*) in the sunlight.

33. Every day, the sirens *blare* (*blares, sew*) to signal emergencies.

34. Every day, the seamstresses *sew* (*sews, blare*) custom designed gowns.

35. Every day, the telephones *ring* (*rings, scurry*) constantly until closing time.

36. Every day, the squirrels *scurry* (*scurries, ring*) under the rose bushes.

37. Every day, the tree *grows* (*grow, plays*) taller and more beautiful.

38. Every day, the theater *plays* (*play, grows*) seven different movies.

39. Every day, the hikers *climb* (*climbs, sparkle*) closer to the mountain’s peak.

40. Every day, the minerals *sparkle* (*sparkles, climb*) in the midday sun.

41. Every day, the girls *giggle* (*giggles, roar*) about the funny cartoons.

42. Every day, the lions *roar* (*roars, giggle*) around dinner time.

43. Every day, the seagull *flies* (*fly, melts*) over the waves.

44. Every day, the wax *melts* (*melt, flies*) onto the table.

45. Every day, the boys *plan* (*plans, overflow*) the next football game.

46. Every day, the rivers *overflow* (*overflows, plan*) the low lands.

47. Every day, the lifeguard *watches* (*watch, sprouts*) the swimmers.

48. Every day, the plant *sprouts* (*sprout, watches*) new leaves.

49. Every day, the gardener *digs* (*dig, drips*) in the flower bed.

50. Every day, the water *drips* (*drip, digs*) into the sink.

51. Every day, the pen *leaks* (*leak, wrinkles*) in my pocket.

52. Every day, the shirt *wrinkles* (*wrinkle, leaks*) in the dryer.

53. Every day, the musicians *tune* (*tunes, rumble*) their instruments.

54. Every day, the trucks *rumble* (*rumbles, tune*) down the highway.

55. Every day, the tooth *aches* (*ache, sing*) when I eat sweets.

56. Every day, the tire *deflates* (*deflate, aches*) a little bit more.

57. Every day, the rose *wilts* (*wilt, swings*) in the hot sunshine.

58. Every day, the door *swings* (*swing, wilts*) shut by the draft.

59. Every day, the chefs *prepare* (*prepares, flicker*) hundreds of meals.

60. Every day, the lights *flicker* (*flickers, prepare*) in the window.

## Abbreviations

AN: Anterior negativity
ERP: Event-related potential
NL: Normal Language
SLI: Specific language impairment
SLI-Persistent, SLI-P: Specific Language Impairment-Persistent
SLI-Recovered, SLI-R: Specific Language Impairment-Recovered

## References

[1] Alt M, Meyers C, Alt PM (2013). Using ratings to gain insight into conceptual development. J Speech Lang Hear Res.

[2] Andreu L, Sanz-Torrent M, Guàrdia-Olmos J (2012). Auditory word recognition of nouns and verbs in children with Specific Language Impairment (SLI). J Commun Disord.

[3] Atchley RA, Rice ML, Betz SK, Kwasny KM, Sereno JA, Jongman A (2006). A comparison of semantic and syntactic event related potentials generated by children and adults. Brain Lang.

[4] Borovsky A, Burns E, Elman JL, Evans JL (2013). Lexical activation during sentence comprehension in adolescents with history of specific language impairment. J Commun Disord.

[5] Catts HW, Fey ME, Tomblin JB, Zhang X (2002). A longitudinal investigation of reading outcomes in children with language impairments. J Speech Lang Hear Res.

[6] Conti-Ramsden G, Durkin K, Simkin Z, Knox E (2009). Specific language impairment and school outcomes. I: identifying and explaining variability at the end of compulsory education. Int J Lang Commun Disord.

[7] Conti-Ramsden G, Clair CS, Pickles A, Durkin K (2012). Developmental trajectories of verbal and nonverbal skills in individuals with a history of specific language impairment: from childhood to adolescence. J Speech Lang Hear Res.

[8] Conti-Ramsden G, Ullman MT, Lum JAG (2015). The relation between receptive grammar and procedural, declarative, and working memory in specific language impairment. Front Psychol.

[9] Culatta B, Page J, Ellis J (1983). Story retelling as a communicative performance screening tool. Lang Speech Hear Serv Sch.

[10] Delorme A, Makeig S (2004). EEGLAB: an open source toolbox for analysis of single-trial EEG dynamics including independent component analysis. J Neurosci Methods.

[11] Dunn LM, Dunn LM (1981). Peabody Picture Vocabulary Test—Revised.

[12] Ellis Weismer S, Evans J, Hesketh LJ (1999). An examination of verbal working memory capacity in children with specific language impairment. J Speech Lang Hear Res.

[13] Fazio BB (1999). Arithmetic calculation, short-term memory, and language performance in children with specific language impairment: a 5-year follow-up. J Speech Lang Hear Res.

[14] Ferreira F, Bailey KGD, Ferraro V (2002). Good-enough representations in language comprehension. Curr Dir Psychol Sci.

[15] Fonteneau E, van der Lely HKJ (2008). Electrical brain responses in language-impaired children reveal grammar-specific deficits. Plos One.

[16] Friederici AD (2002). Towards a neural basis of auditory sentence processing. Trends Cogn Sci.

[17] Friedrich M, Friederici AD (2010). Maturing brain mechanisms and developing behavioral language skills. Brain Lang.

[18] Gouvea AC, Phillips C, Kazanina N, Poeppel D (2010). The linguistic processes underlying the P600. Lang Cogn Process.

[19] Grier JB (1971). Nonparametric indexes for sensitivity and bias: computing formulas. Psychol Bull.

[20] Haebig E, Kaushanskaya M, Ellis Weismer S (2015). Lexical processing in school-age children with autism spectrum disorder and children with specific language impairment: the role of semantics. J Autism Dev Disord.

[21] Hahne A, Eckstein K, Friederici AD (2004). Brain signatures of syntactic and semantic processes during children’s language development. J Cogn Neurosci.

[22] Hays WL (1994). Inferences about population means. Statistics..

[23] Hesketh A, Conti-Ramsden G (2013). Memory and language in middle childhood in individuals with a history of specific language impairment. Plos One.

[24] Holcomb PJ, Coffey S a, Neville H (1992). Visual and auditory sentence processing: a developmental analysis using event-related brain potentials. Dev Neuropsychol.

[25] Holcomb PJ, Coffey SA, Neville HJ (1992). Visual and auditory sentence processing: a developmental analysis using event‐related brain potentials. Dev Neuropsychol.

[26] Jung TP, Makeig S, Westerfield M, Townsend J, Courchesne E, Sejnowski TJ (2000). Removal of eye activity artifacts from visual event-related potentials in normal and clinical subjects. Clin Neurophysiol.

[27] Jurcak V, Tsuzuki D, Dan I (2007). 10/20, 10/10, and 10/5 systems revisited: their validity as relative head-surface-based positioning systems. Neuroimage.

[28] Karmiloff-Smith A (2009). Nativism versus neuroconstructivism: rethinking the study of developmental disorders. Dev Psychol.

[29] Kim A, Osterhout L (2005). The independence of combinatory semantic processing: evidence from event-related potentials. J Mem Lang.

[30] Kutas M, Federmeier KD (2011). Thirty years and counting: finding meaning in the N400 component of the event-related brain potential (ERP). Annu Rev Psychol.

[31] Kutas M, Hillyard SA (1980). Reading senseless sentences: brain potentials reflect semantic incongruity. Science.

[32] Leonard LB (2014). Children with specific language impairment.

[33] Leonard LB, Eyer JA, Bedore LM, Grela BG (1997). Three accounts of the grammatical morpheme difficulties of English-speaking children with specific language impairment. J Speech Lang Hear Res.

[34] Leonard LB, Camarata SM, Brown B, Camarata MN (2004). Tense and agreement in the speech of children with specific language impairment: patterns of generalization through intervention. J Speech Lang Hear Res.

[35] Leonard LB, Ellis Weismer S, Miller CA, Francis DJ, Tomblin JB, Kail RV (2007). Speed of processing, working memory, and language impairment in children. J Speech Lang Hear Res.

[36] Leonard LB, Miller CA, Finneran DA (2009). Grammatical morpheme effects on sentence processing by school-aged adolescents with specific language impairment. Lang Cogn Process.

[37] Leslie L, Cladwell J (2000). Qualitative Reading Inventory-3.

[38] Linebarger MC, Schwartz MF, Saffran EM (1983). Sensitivity to grammatical structure in so-called agrammatic aphasics. Cognition.

[39] Locke JL (1994). Gradual emergence of developmental language disorders. J Speech Lang Hear Res.

[40] Lopez-Calderon J, Luck SJ (2014). ERPLAB: an open-source toolbox for the analysis of event-related potentials. Front Hum Neurosci.

[41] Lopez-Calderon, J., & Luck, S. J. ERPLAB Toolbox (1.1.0). (2010). Retrieved from http://erpinfo.org/erplab. Accessed 10 Sept 2015.

[42] Luck SJ (2014). An introduction to the event-related potential technique.

[43] Marton K (2008). Visuo-spatial processing and executive functions in children with specific language impairment. Int J Lang Commun Disord.

[44] Marton K, Eichorn N (2014). Interaction between working memory and long-term memory. Zeitschrift Für Psychologie.

[45] Miller CA, Leonard LB, Finneran D (2008). Grammaticality judgements in adolescents with and without language impairment. Int J Lang Commun Disord.

[46] Neville H, Coffey S a, Holcomb PJ, Tallal P (1993). The neurobiology of sensory and language processing in language-impaired children. J Cogn Neurosci.

[47] Newcomer P, Hammill D (1988). Test of Language Development-Primary.

[48] Nieuwland MS, Martin AE, Carreiras M (2013). Event-related brain potential evidence for animacy processing asymmetries during sentence comprehension. Brain Lang.

[49] Oldfield RC (1971). The assessment and analysis of handedness: the Edinburgh inventory. Neuropsychologia.

[50] Osterhout L, Holcomb PJ (1992). Event-related brain potentials elicited by syntactic anomaly. J Mem Lang.

[51] Osterhout L, Holcomb PJ, Swinney DA (1994). Brain potentials elicited by garden-path sentences: evidence of the application of verb information during parsing. J Exp Psychol Learn Mem Cogn.

[52] Osterhout L, Kim A, Kuperberg G, Spivey M, Joannisse M, McRae K (2012). The neurobiology of sentence comprehension. Cambridge handbook of psycholinguistics.

[53] Pakulak E, Neville H (2010). Proficiency differences in syntactic processing of monolingual native speakers indexed by event-related potentials. J Cogn Neurosci.

[54] Park J, Miller CA, Mainela-Arnold E (2015). Processing speed measures as clinical markers for children with language impairment. J Speech Lang Hear Res.

[55] Phillips C, Kazanina N, Abada SH (2005). ERP effects of the processing of syntactic long-distance dependencies. Cogn Brain Res.

[56] Plante E, Van Petten C, Senkfor AJ (2000). Electrophysiological dissociation between verbal and nonverbal semantic processing in learning disabled adults. Neuropsychologia.

[57] Purdy JD, Leonard LB, Weber-Fox C, Kaganovich N (2014). Decreased sensitivity to long-distance dependencies in children with a history of specific language impairment: electrophysiological evidence. J Speech Lang Hear Res.

[58] Redmond SM, Rice ML (2001). Detection of irregular verb violations by children with and without SLI. J Speech Lang Hear Res.

[59] Rice ML, Hoffman L (2015). Predicting vocabulary growth in children with and without specific language impairment: a longitudinal study from 2;6 to 21 years of age. J Speech Lang Hear Res.

[60] Rice ML, Wexler K (1996). Toward tense as a clinical marker of specific language impairment in English-speaking children. J Speech Lang Hear Res.

[61] Rice ML, Wexler K, Redmond SM (1999). Grammaticality judgements of extended optional infinitive grammar: evidence from English-speaking children with specific language impairment. J Speech Lang Hear Res.

[62] Rice ML, Redmond SM, Hoffman L (2006). Mean length of utterance in children with specific language impairment and in younger control children shows concurrent validity and stable and parallel growth trajectories mean length of utterance in children and parallel growth trajectories. J Speech Lang Hear Res.

[63] Rice ML, Hoffman L, Wexler K (2009). Judgments of omitted BE and DO in questions as extended finiteness clinical markers of specific language impairment (SLI) to 15 years: a study of growth and asymptote. J Speech Lang Hear Res.

[64] Sabisch B, Hahne A, Glass E, von Suchodoletz W, Friederici AD (2006). Lexical-semantic processes in children with specific language impairment. Neuroreport.

[65] Sabisch B, Hahne CA, Glass E, von Suchodoletz W, Friederici AD (2009). Children with specific language impairment: the role of prosodic processes in explaining difficulties in processing syntactic information. Brain Res.

[66] Scarborough HS, Dobrich W (1990). Development of children with early language delay. J Speech Lang Hear Res.

[67] Schneider JM, Abel AD, Ogiela DA, Middleton A, Maguire MJ (2016). Developmental differences in beta and theta power during sentence processing. Dev Cogn Neurosci.

[68] Semel E, Wiig EH, Secord W (1995). Clinical evaluation of language fundamentals. (T. P. Corporation, Ed.).

[69] Severens E, Jansma BM, Hartsuiker RJ (2008). Morphophonological influences on the comprehension of subject-verb agreement: an ERP study. Brain Res.

[70] Sheng L, McGregor KK (2010). Lexical-semantic organization in children with specific language impairment. J Speech Lang Hear Res.

[71] Sitnikova T, Holcomb PJ, Kuperberg GR, Shipley TF, Zacks JM (2008). Neurocognitive mechanisms of human comprehension. Understanding events: how humans see, represent, and act on events.

[72] Steinhauer K, Drury JE (2012). On the early left-anterior negativity (ELAN) in syntax studies. Brain Lang.

[73] Tanner D, Van Hell JG (2014). ERPs reveal individual differences in morphosyntactic processing. Neuropsychologia.

[74] Tomblin JB, Records NL, Zhang X (1996). A system for the diagnosis of specific language impairment in kindergarten children. J Speech Lang Hear Res.

[75] Tomblin JB, Records NL, Buckwalter P, Zhang X, Smith E, O’Brien M (1997). Prevalence of specific language impairment in kindergarten children. J Speech Lang Hear Res.

[76] Ullman MT, Pierpont EI (2005). Specific language impairment is not specific to language: the procedural deficit hypothesis. Cortex.

[77] van de Meerendonk N, Kolk HHJ, Vissers CTWM, Chwilla DJ (2010). Monitoring in language perception: mild and strong conflicts elicit different ERP patterns. J Cogn Neurosci.

[78] Wallace GL, Hammill D (1994). Comprehensive receptive and expressive vocabulary test.

[79] Weber-Fox C, Hampton A (2008). Stuttering and natural speech processing of semantic and syntactic constraints on verbs. J Speech Lang Hear Res.

[80] Weber-Fox C, Leonard LB, Hampton Wray A, Tomblin JB (2010). Electrophysiological correlates of rapid auditory and linguistic processing in adolescents with specific language impairment. Brain Lang.

[81] Wechsler D (1991). Wechsler Intelligence Scale for Children—Third Edition.

[82] Wittke K, Spaulding TJ, Schechtman CJ (2013). Specific language impairment and executive functioning: parent and teacher ratings of behavior. Am J Speech Lang Pathol.

[83] Wulfeck B, Bates E, Krupa-Kwiatkowski M, Saltzman D (2004). Grammaticality sensitivity in children with early focal brain injury and children with specific language impairment. Brain Lang.

